# Molecular Dynamics Study of Bending Deformation of Mo_2_Ti_2_C_3_ and Ti_4_C_3_ (MXenes) Nanoribbons

**DOI:** 10.3390/molecules29194668

**Published:** 2024-10-01

**Authors:** Vadym Borysiuk, Iakov A. Lyashenko, Valentin L. Popov

**Affiliations:** 1 Department of System Dynamics and Friction Physics, Institute of Mechanics, Technische Universität Berlin, 10623 Berlin, Germany; i.liashenko@tu-berlin.de (I.A.L.); v.popov@tu-berlin.de (V.L.P.); 2 Department of Computerized Control Systems, Faculty of Electronics and Information Technology, Sumy State University, 40007 Sumy, Ukraine; 3 Department of Theoretical and Applied Mechanics, Samarkand State University, Samarkand 140104, Uzbekistan; 4 Center of Advanced Studies in Mechanics, Tribology, Bio- and Nanotechnologies, Samarkand State University, Samarkand 140104, Uzbekistan

**Keywords:** molecular dynamics, 2D materials, MXenes, double transition metal carbides, nanoribbon, bending rigidity, central deflection

## Abstract

We report a computational study of the bending deformation of two-dimensional nanoribbons by classical molecular dynamics methods. Two-dimensional double transition metal carbides, together with monometallic ones, belong to the family of novel nanomaterials, so-called MXenes. Recently, it was reported that within molecular dynamics simulations, Ti_4_C_3_ MXene nanoribbons demonstrated higher resistance to bending deformation than thinner Ti_2_C MXene and other two-dimensional materials, such as graphene and molybdenum disulfide. Here, we apply a similar method to that used in a previous study to investigate the behavior of Mo_2_Ti_2_C_3_ nanoribbon under bending deformation, in comparison to the Ti_4_C_3_ sample that has a similar structure. Our calculations show that Mo_2_Ti_2_C_3_ is characterized by higher bending rigidity at $D_{{\mathrm{Ti}}_{2}{\mathrm{Mo}}_{2}C_{3}}\approx 92.15$ eV than monometallic Ti_4_C_3_ nanoribbon at $D_{{\mathrm{Ti}}_{4}C_{3}}\approx 72.01$ eV, which has a similar thickness. Moreover, approximately the same magnitude of critical central deflection of the nanoribbon before fracture was observed for both Mo_2_Ti_2_C_3_ and Ti_4_C_3_ samples, $w_{c}\approx 1.7$ nm, while Mo_2_Ti_2_C_3_ MXene is characterized by almost two times higher critical value of related external force.

## 1. Introduction

Two-dimensional (2D) crystals are a relatively new class of structure that includes several families of various materials. The most popular and well-known among 2D crystals is graphene—an atomically thin layer of carbon atoms with hexagonal structure and covalent bonds, discovered in 2004 [1]. Other popular representatives of 2D crystals are 2D boron nitride [2], 2D molybdenum disulfide MoS_2_ [3], 2D transition metal dichalcogenides (2D TMDs), and other materials [4]. Since their discovery, two-dimensional materials have opened new possibilities to significantly improve nanoelectronics, due to their ability to be integrated into devices with atomic-scale precision, leading to the development of smaller, faster, and more efficient electronic components. Thus, each of the 2D materials has unique physical properties that define their potential application. For instance, graphene has exceptional electron mobility, mechanical strength, and thermal conductivity. Its application in nanoelectronics includes the development of high-speed transistors, flexible transparent conductive films, and advanced sensors [5,6]. Graphene-based transistors, for instance, can operate at higher frequencies compared to traditional silicon-based transistors, enabling faster data processing and communication [7,8].

In 2011, the family of 2D materials became even bigger, as a new group of crystals—transition metals carbides and nitrides (as well as carbonitrides) with general chemical formula M_n+1_X_n_ (M is an early transition metal, and X is carbon C and/or nitrogen N) called MXenes—were discovered [9]. MXenes are synthesized from bulk precursors, so-called MAX-phases (nanolaminated materials with general chemical formula M_n+1_AX_n_, where M, as above, is an early transition metal, A is an element from group IIIA or IVA (or group 13 or 14), and X is carbon C and/or nitrogen N, and n = 1, 2, or 3) by etching a layer of element A atoms from MAX phases [10,11].

Together with unique electrochemical properties [12,13,14], MXenes exhibit exceptional mechanical properties [15,16], including high strength, flexibility, and tunability, making them highly attractive for applications in nanoelectronics. Their ability to withstand mechanical deformation while maintaining electronic functionality opens up new possibilities for the development of flexible, durable, and high-performance electronic devices [17,18].

Additionally, the layered structure of MXenes contributes to their mechanical stability and flexibility. The layers can slide relative to each other, providing an intrinsic mechanism for accommodating strain and preventing fracture. This characteristic is particularly beneficial for creating multilayered or composite structures that combine MXenes with other materials, resulting in devices that are both mechanically robust and electrically superior. The integration of MXenes into nanoelectromechanical systems (NEMSs), leverages their mechanical properties to enhance device performance and longevity [17,18].

The flexibility and mechanical robustness of MXenes are further enhanced by their unique surface chemistry. MXenes can be functionalized with various surface groups, such as hydroxyl, oxygen, and fluorine, which not only modify their electronic properties but also influence their mechanical behavior. This tunability allows researchers to design MXenes with tailored mechanical properties to meet specific application requirements. For instance, by adjusting the surface chemistry, MXenes can achieve an optimal balance between flexibility and strength, making them ideal for applications in nanoelectronics where mechanical reliability is paramount.

One of the important properties of nanomaterials that defines their use in nanoelectromechanical system devices is bending or flexural rigidity [19,20]. It characterizes the ability of a particular sample to resist bending deformation and maintain its flexibility and elasticity. However, the experimental measurement of the mechanical properties of nanomaterials requires special facilities and represents a serious challenge due to the small sizes of the samples (see for example [21,22,23]). Therefore, mechanical properties of nanomaterials are also studied by computational [15,16,17,18,19,20] and theoretical [24,25,26] methods. Thus, one of the illustrative examples of such study is the calculation of bending rigidity of graphene nanoribbons (GNRs) by classical molecular dynamics simulations [20]. The developed approach also allowed the authors to propose a model for an ultrasensitive pressure sensor based on GNRs, and also suggested further applications of graphene nanoribbons in NEMSs devices in general [27].

A similar approach was recently adopted to study the bending properties of the Ti_n+1_C_n_ MXene nanoribbons [19]. The performed calculations confirmed the assumption that Ti_n+1_C_n_ samples are characterized by higher bending rigidity than graphene, due to the higher thickness of MXene nanoribbons, as well as the increasing of bending rigidity with the growth of the thickness of the samples. Moreover, recent discoveries of the ordered double transition metal carbides [28,29] opened a new way of controlling and tuning the electronic properties of MXenes by altering the outer metal layer in M_n+1_X_n_ sheets. Thus, after the synthesis of double transition metals, MXene Mo_2_Ti_2_C_3_ was reported [29], which naturally opened a question of how the changing of two outer layers of titanium in Ti_4_C_3_ to molybdenum affects the flexural properties of MXene nanoribbons. In this paper, we report the comparative analysis of the bending properties of Mo_2_Ti_2_C_3_ and Ti_4_C_3_ by classical molecular dynamics simulations that were performed using a previously developed computational scheme of numerical experiments on bending of Ti_n+1_C_n_ MXene nanoribbons [19]. As no experiments on bending rigidity of pristine Mo_2_Ti_2_C_3_ and Ti_4_C_3_ nanoribbons have been reported by now, our study may provide the first insights on how mechanical properties of MXenes can be affected by replacing monometallic Ti sample by (Mo, Ti) double transition metal carbide.

## 2. Model

**Figure 1:**
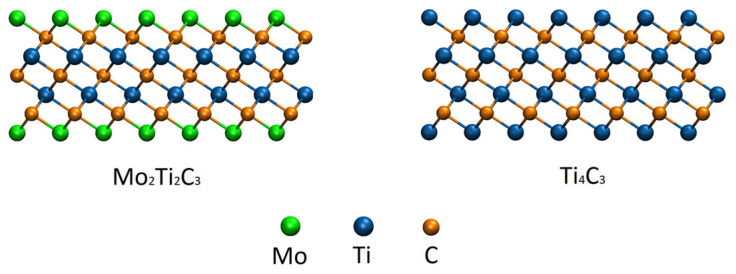
Atomistic configurations of the studied Mo_2_Ti_2_C_3_ (**left panel**) and Ti_4_C_3_ (**right panel**) samples. Related colors of the atoms are shown in the figure.

**Figure 2:**
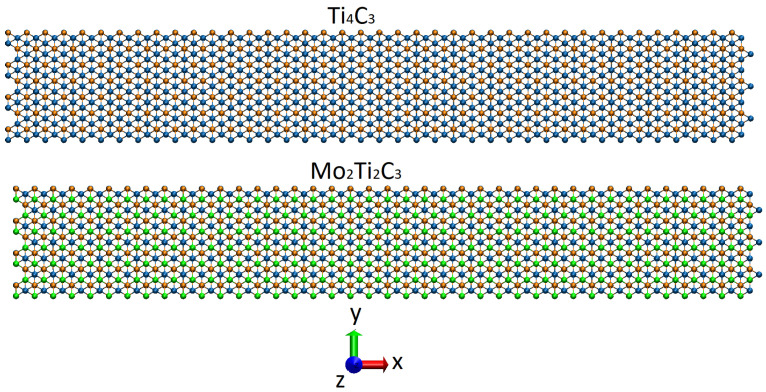
Snapshots (top view in Cartesian coordinate system XYZ) of the initial configuration of the studied Ti_4_C_3_ (**top panel**) and Mo_2_Ti_2_C_3_ (**bottom panel**) nanoribbons. The colors of atoms are the same as in Figure 1.

We followed a previous study on the bending rigidity of Ti_n+1_C_n_ MXene nanoribbons [19], where preliminary results on the bending of Ti_4_C_3_ nanoribbon were reported. In our simulations, we consider two different MXene nanoribbons, Mo_2_Ti_2_C_3_ and Ti_4_C_3_. The chemical composition of the considered samples was chosen due to the already developed MD approach for the simulation of Ti_n+1_C_n_ samples [15] and similar structures of Mo_2_Ti_2_C_3_ and Ti_4_C_3_ MXenes [28,29]. As it is reported in [29], the double transition metal Mo_2_Ti_2_C_3_ is characterized by almost equivalent interatomic distances between Mo-C and Ti-C atoms, similar lattice parameters, and the same sample thickness as Ti_4_C_3_ MXene. The structures of both Mo_2_Ti_2_C_3_ and Ti_4_C_3_ MXenes and the general view of the studied nanoribbons are shown in Figure 1 and Figure 2, respectively (all snapshots of atomistic configurations of the samples were prepared with visual molecular dynamics software version 1.9 [30]).

Moreover, with the assumption that bending rigidity depends on the thickness *h* of the sample being $\propto h^{3}$ [19], simulations of the thickest (seven atomic layers) M_4_X_3_ samples under bending deformation may provide the approximal value of maximal bending rigidity that can be expected among (Mo,Ti)_n+1_C_n_ and Ti_n+1_C_n_ MXenes.

It is worth to note that several approaches for MD simulations of MXenes were reported in the literature so far (see for example [15,16,31,32]). The most precise and yet most complicated approach employs ReaxFF interatomic potential [33]. Such an approach allowed us to obtain important data on chemical reactions and phase transitions within MXenes, and to study their mechanical properties as well [16,33]. However, ReaxFF can be straightforwardly used for simulations of certain MXenes only, and its application for Mo_2_Ti_2_C_3_ requires additional parametrization, which is a rather hard challenge [33]. Therefore, in our simulations, we adopted a previously developed approach that is based on combinations of interatomic potentials [15]. The full description of the developed model is provided in the original paper; therefore, here we focused on the adjustments that were introduced in the model to adapt for simulations of Mo_2_Ti_2_C_3_ MXene. Following the assumption that interactions between titanium atoms in Ti_n+1_C_n_ MXenes can be described within embedded atom method (EAM) [15], we suppose that EAM potential can also be used to describe interactions between molybdenum atoms in Mo_2_Ti_2_C_3_ samples in a similar manner, as EAM potential is already parametrized for molybdenum [34]. Moreover, with this assumption, interactions between Ti and Mo atoms in Mo_2_Ti_2_C_3_ MXene sample can be described with an EAM alloy model for Ti-Mo [34]. As in Mo_2_Ti_2_C_3,_ Mo-C and Ti-C bonds have almost similar lengths [29], interatomic forces between both metals and carbon atoms are calculated in the same way as forces between titanium and carbon in Ti_4_C_3_ [19]. This is a rough approximation, which makes our model rather qualitative; nevertheless, it makes it possible to obtain preliminary data on flexural properties of double metals Mo_2_Ti_2_C_3_ MXene, without the complicated parametrization of other interatomic potentials.

**Figure 3:**
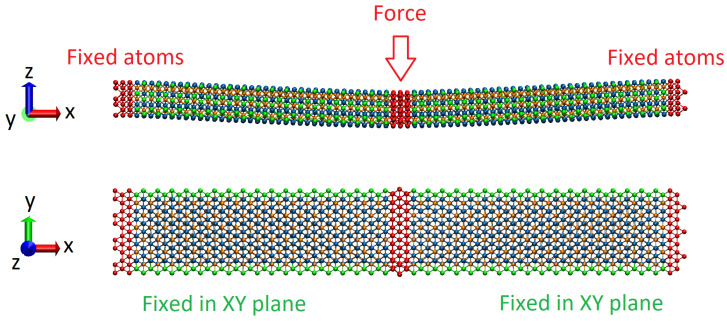
Schematics of the experiments on the bending of Mo_2_Ti_2_C_3_ and Ti_4_C_3_ nanoribbons in Cartesian coordinate system XYZ. Fixed atoms, on the edges of the sample and central atoms to which external bending load is applied, are shown in red color. Constrained atoms across long edges are denoted in green.

In our experiments on bending, we followed an already existing methodic that was proposed for studying the flexural properties of graphene nanoribbons [20], and which was further adopted for the calculation of the bending rigidity of Ti_n+1_C_n_ MXenes [19]. We consider Mo_2_Ti_2_C_3_ and Ti_4_C_3_ nanoribbons with sizes of 1.7 nm × 12.0 nm located in a Cartesian coordinate box with periodic boundary conditions in X and Y direction, and free boundary condition in *Z*, as shown by schematics in Figure 3. For the same reason, as in [19], we applied similar constraints to the boundary atoms across long edges of the nanoribbons, not allowing them to relax in the XY plane during bending.

Thus, by applying the external force *F*, as is shown in Figure 3, and measuring the corresponding central deflection of nanoribbon w, it is possible to calculate the bending rigidity *D* of the sample, from the well-known equation [19,20]:

(1) $$\frac{F}{w}=\frac{D}{\beta L^{2}}$$

where *L* is the length of the nanoribbon and *β* is the coefficient in the range from 0.0056 to 0.00725 for a nanoribbon length-to-width ratio from 1 to ∞, respectively.

In our simulation, we adopted an in-house code, developed for the study of mechanical properties and thermal stability of Ti_n+1_C_n_ MXenes [15,19,35,36]. The code was implemented on a graphics processing unit (GPU) for parallel calculations. The calculations were performed on an NVIDIA Tesla P100 GPU (NVIDIA Corporation, Santa Clara, CA, USA). Other details of the simulation setup can be found in [19]. The obtained results are described in the following section.

## 3. Results

**Figure 4:**
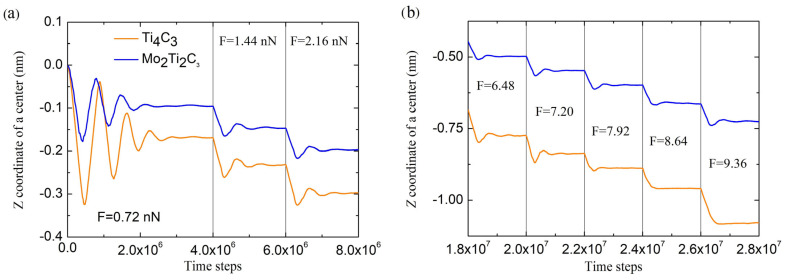
Time dependencies of the *Z* coordinate of centers of the samples. Panel (**a**) shows the behavior of the samples in the initial stage of the experiment for the first three steps of loading, while panel (**b**) demonstrates the behavior in the middle of simulations.

As described above, the simulations of the bending deformation of MXene nanoribbons were performed by applying external force in the –Z direction to the central part of the sample (see Figure 3). Applications of the external load results in a deflection of the nanoribbon from the initial configuration and its bending. Similar to the simulations described in [19,20], we performed the simulation by gradually increasing the force applied to the sample and analyzing its behavior. During the experiments, the time dependencies of the immediate position of the nanoribbon center and other data needed for calculations of bending parameters were recorded. The typical behavior of the samples under an external bending load is shown in Figure 4.

Presented in Figure 4, data show that at the beginning of experiments, the application of the external force of the smallest magnitude *F*_0_ (equivalent to 0.72 nN) initiates damping oscillations of both nanoribbons (see opening part of the dependencies in Figure 4a), after which the sample deflects from the original position. After applying forces of larger magnitudes (with an increment $\Delta F=F_{0}$), central deflection w was measured after slight damping oscillations when samples reached the stationary configuration. In our experiments, samples were allowed to reach a stationary position for 2 × 10^6^ time steps (4 × 10^6^ time steps for initial indentation, as shown in Figure 4a). The central deflection was measured by time averaging of the *Z* coordinate of the central part of the sample, to which the external force is applied (see Figure 3) over the last 5 × 10^5^ time steps during each force increment. Figure 4b shows that each force increment ΔF resulted in approximately constant changes in each sample’s increment of central deflection Δw, which suggests that in this part of experiment, nanoribbons are bending in an almost linear regime.

**Figure 5:**
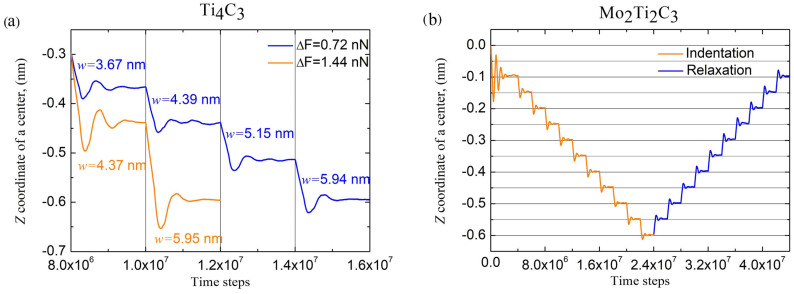
(**a**) Time dependencies of *Z* coordinate of Ti_4_C_3_ nanoribbon during bending with different force increments, ΔF and 2ΔF (magnitudes of central deflection and force increments are shown in the figure). Time dependencies of *Z* coordinate of Mo_2_Ti_2_C_3_ nanoribbons during indentation and relaxation (**b**).

To illustrate the behavior of the samples in elastic regimes of bending deformation, we performed additional indentation of the Ti_4_C_3_ sample with the doubled force increment 2ΔF, as well as the gradual relaxation of Mo_2_Ti_2_C_3_ nanoribbon. Obtained data are shown in Figure 5.

As it follows from the data presented in Figure 5a, the application of a two-times larger force increment 2ΔF to Ti_4_C_3_ sample results in approximately two times larger increments of related central deflection $2\Delta w_{{\mathrm{Ti}}_{4}C_{3}}$ compared to the initial conditions of the experiments. The presented dependencies are also characterized by a larger amplitude of damping oscillations after each increase in the bending force. Figure 5b shows the behavior of the Mo_2_Ti_2_C_3_ sample during the first 10 steps of indentation (increasing of the applied force F with constant increment ΔF) and following relaxation (decreasing of F with constant increment −ΔF) to the previous position. As it follows in the figure, consecutive relaxation of the applied force results in decreases in the central deflection by almost the same increment $\Delta w_{{\mathrm{Mo}}_{2}{\mathrm{Ti}}_{2}C_{3}}$ as during the indentation. Therefore, the presented dependence illustrates the reversibility of the performed experiments within the elastic mode of the bending deformation of nanoribbons.

**Figure 6:**
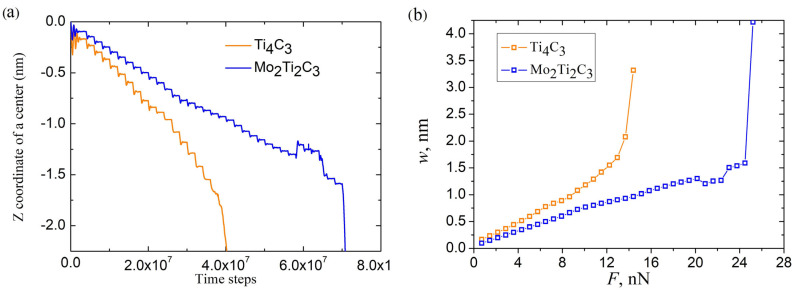
(**a**) Time dependencies of *Z* coordinate of Ti_4_C_3_ and Mo_2_Ti_2_C_3_ nanoribbons during the whole experiment. (**b**) Corresponding load curves w(F) for Ti_4_C_3_ and Mo_2_Ti_2_C_3_ nanoribbons. The final points on the dependencies plotted in panel (**b**) do not relate to corresponding magnitude of central deflection of nanoribbons; these points were measured after the fracture of nanoribbons in the certain time moments, according to computational algorithm and plotted in the figure to illustrate the fracturing of the samples.

Further in our experiments, the application of the increasing external force continued until the complete fracture of the nanoribbon. The total time dependencies of the *Z* coordinate of nanoribbon centers and the load curves w(F) for both samples are shown in Figure 6a and Figure 6b, respectively.

The fracture of nanoribbon is indicated by the sharp drop in *Z*(*t*) dependencies to large negative values of *Z* (see Figure 6a). As it follows from the figure, both samples are characterized by a close magnitude of critical deflection $w_{c}\approx 1.7$ nm before the destruction of the nanoribbon. At the same time, as Figure 6b shows, the fracturing of Mo_2_Ti_2_C_3_ nanoribbon was observed at an almost two times larger magnitude of the critical external force $F_{c}$ (note that there were significantly more indentation steps and correspondingly more time steps in simulations for Mo_2_Ti_2_C_3_ nanoribbon before fracturing, as shown in Figure 6a).

**Figure 7:**
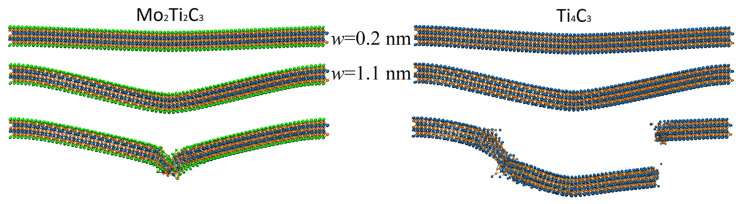
Examples of typical atomistic configuration of Mo_2_Ti_2_C_3_ (**left panel**) and Ti_4_C_3_ (**right panel**) nanoribbons at different magnitudes of central deflection. The bottom panels show the plastic deformation of the Mo_2_Ti_2_C_3_ sample and the fracture of the Ti_4_C_3_ nanoribbon.

It is important to note that nonlinear modes of bending, related to plastic deformation and partial destruction of the samples, are also clearly visible from the data presented in Figure 6. The mentioned modes of bending relate to the part of the dependencies where the growth of the external force results in increases in the corresponding increments of central deflection Δw or in decreases in the current magnitude of central deflection w(F) presented in Figure 6b. The latter case relates to the plastic deformation and partial destruction of the sample, which may lead to the local rearrangement of atoms and relaxation of mechanical stresses. Moreover, it is worth noting that the nonlinear dependence of central deflection on the applied load w(F) may also be caused by the axial extension effect [37]. An example of atomistic configuration of Mo_2_Ti_2_C_3_ nanoribbon with clearly visible plastic deformation and partial destruction of the central part is shown in Figure 7.

Figure 7 shows a typical atomistic configuration of studied samples in different modes of bending deformation. The top panels show configurations of the samples at central deflection w≈0.2 nm, which relate to the elastic mode of bending, corresponding to linear parts of the dependencies of central deflection on external force w(F), as shown in Figure 6b. The middle panels of Figure 7 depict the configuration of the samples at larger central deflection w≈1.1 nm. At these conditions, force increment ΔF no longer leads to constant increments of central deflection Δw; however, no plastic deformation or fracture of the samples is observed in atomistic configurations, and samples can be relaxed to initial configuration. The bottom panels of Figure 7 show the fracture of the samples. Notably, samples are characterized by different fracture dynamics. Thus, Mo_2_Ti_2_C_3_ nanoribbon is characterized by severe plastic deformation in the central area where the external force is applied, following the destruction of the MXene crystal structure and the fracture of the nanoribbon. At the same time, fracture dynamics of Ti_4_C_3_ nanoribbon are more typical for brittle materials, with the destruction of the sample in several areas, and following the formation of fragments that preserve the two-dimensional structure of Ti_4_C_3_ MXene. Such behavior of Ti_4_C_3_ MXenes was already observed in our earlier MD simulations [19]. The bending and fracture dynamics of both nanoribbons are also shown in Supplementary Videos S1 and S2.

**Figure 8:**
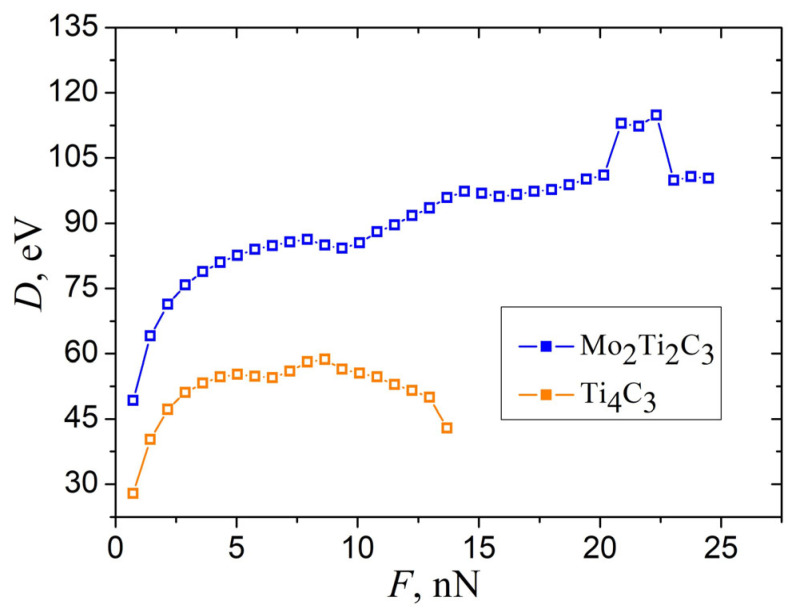
The dependence of bending rigidity *D* of Mo_2_Ti_2_C_3_ and Ti_4_C_3_ samples, calculated from the data shown in Figure 6b, according to Equation (1).

Performing simulations and recorded data allowed us to calculate elastic parameters that characterize the bending properties of MXene nanoribbons. However, the straightforward installation of obtained w(F) dependence (shown in Figure 6b) in Equation (1) leads to the bending rigidity *D* of the samples, as presented in Figure 8.

As it can be seen from the figure, the calculated *D* strongly depends on applied force even in the region of small magnitudes of central deflections. This makes it hard to obtain the exact value of the elastic parameters that characterize the bending properties of MXene nanoribbons. However, this feature can be resolved by analyzing the dependencies shown in Figure 4a and Figure 6.

**Figure 9:**
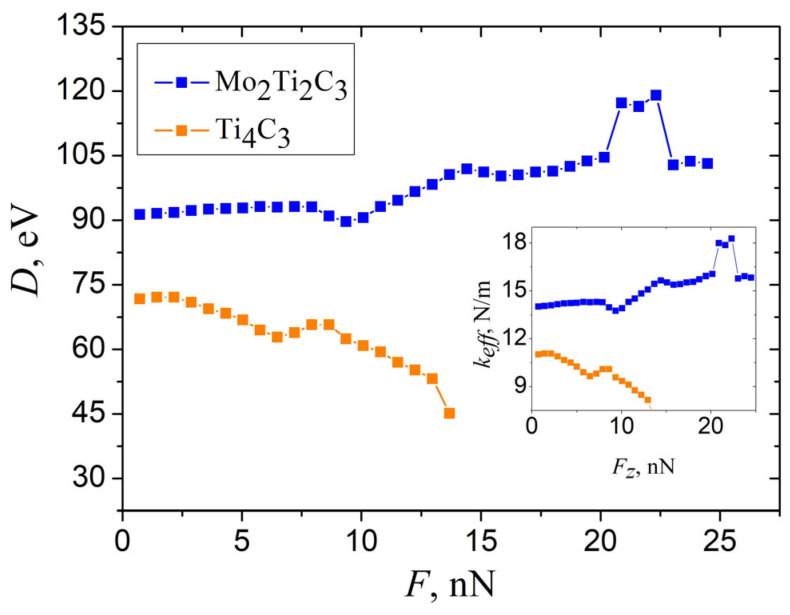
The dependence of bending rigidity *D* of Mo_2_Ti_2_C_3_ and Ti_4_C_3_ samples, calculated from the data shown in Figure 6b, according to Equation (1), taking into account of the pre-tension of the sample. Inset shows related dependencies of effective spring constant $k_{eff}=F/w$.

As can be seen in Figure 4a, the first increment of a central deflection $\Delta w_{1}$ in the opening part of dependencies has a larger magnitude than the following deflection increments for both samples. Moreover, the system requires more simulation time to reach the stationary configuration, and related damped oscillations of the nanoribbon last longer, compared to the next steps of indentation. This may be explained by the presence of initial deflection $w_{0}$ of the samples and related initial stresses (pre-tension) due to the applied boundary conditions in the *XY* plane. It is worth to mention that the pre-tension of the samples is often observed during the calculation of mechanical parameters in MD simulations [16]. The pre-tension of the samples can be taken into account by introducing the correction $w_{0}$ to measure deflection w for each sample correspondingly. The magnitude of $w_{0}$ can be estimated as a residual part of the first increment $\Delta w_{1}$ after subtracting the deflection increment averaged over the next few steps of indentation, $w_{0\;}$=$\;\Delta w_{1}-\langle \Delta w_{2-5}\rangle$. Thus, magnitudes of initial deflection, calculated according to this assumption, equal $w_{0}=0.044$ nm and 0.103 nm for Mo_2_Ti_2_C_3_, and Ti_4_C_3_ nanoribbons, respectively. Dependencies of bending rigidity D(F) on external force, calculated from Equation (1) after introducing of such correction, are shown in Figure 9.

As it follows from the figure, dependence D(F) calculated by taking into account of the initial deflection of Mo_2_Ti_2_C_3_ sample almost independently of applied force in the elastic region of bending deformation F<7 nN and has an almost constant value of approximately D≈92.15 eV. At the same time, Ti_4_C_3_ nanoribbon is characterized by a shorter linear mode compared to Mo_2_Ti_2_C_3_; however, it is possible to estimate the approximate value of bending rigidity D≈72.01 eV from the initial part of related dependence.

## 4. Discussion

We reported the results of classical molecular dynamics simulations of bending deformation of Mo_2_Ti_2_C_3_ and Ti_4_C_3_ MXene nanoribbons. Our experiments revealed that bending rigidity obtained from the linear part of load curve w(F) for double transition metal carbide Mo_2_Ti_2_C_3_ $D_{{\mathrm{Mo}}_{2}{\mathrm{Ti}}_{2}C_{3}}\approx 92.15$ eV is notably higher than the related parameter of Ti_4_C_3_ MXene $D_{{\mathrm{Ti}}_{4}C_{3}}\approx 72.01$ eV. Moreover, a complete fracture of Mo_2_Ti_2_C_3_ sample was observed at almost twice the larger magnitude of external force $F_{{\mathrm{Mo}}_{2}{\mathrm{Ti}}_{2}C_{3}}\approx 24.48$ nN, compared to $F_{{\mathrm{Ti}}_{4}C_{3}}\approx 12.96$ nN for Ti_4_C_3_. Also, linear mode of bending of Mo_2_Ti_2_C_3_ sample was observed within a wider range of magnitudes of external force, compared to Ti_4_C_3_. This situation may be caused mainly by a higher mass of the molybdenum, compared to titanium, which makes Mo_2_Ti_2_C_3_ sample more massive than Ti_4_C_3_, and it requires a larger force magnitude for acceleration. Another reason for such behavior may be the different interactions between metal atoms within Mo layers compared to Ti layers, as within our model, bonds between Mo and C have the same strength and length as bonds between Ti and C. The latter are features of the developed approach; however, may be the reason for almost the same magnitude of critical central deflection before fracture $w_{c}\approx 1.7$ nm for both samples.

It is important to note that in our calculations, we used empirically chosen parameters to describe interatomic forces between atoms within Mo_2_Ti_2_C_3_ sample; therefore, obtained parameters need further validation by experimental measurements or more accurate computations by MD or DFT methods. Nevertheless, our study provides qualitative insights into the processes that occur at atomistic levels during the bending deformation of 2D nanoribbons. Also, the obtained data may become useful in further theoretical and experimental studies of mechanical properties of 2D materials and their possible applications in the design of NEMSs.

## Data Availability

The original contributions presented in the study are included in the article, further inquiries can be directed to the corresponding author.

## Supplementary Materials

The following supporting information can be downloaded at: https://www.mdpi.com/article/10.3390/molecules29194668/s1, Video S1: Animation of the bending deformation and fracture dynamics of Ti_4_C_3_ nanoribbon. Video S2: Animation of the bending deformation and fracture dynamics of Mo_2_Ti_2_C_3_ nanoribbon.
